# Body composition analysis to determine gender specific physical fitness equations in a cohort of Saudi population

**DOI:** 10.12669/pjms.304.4974

**Published:** 2014

**Authors:** Muhammad Iqbal, Khalid A Al-Regaiey, Shafiq Ahmad, Laila Al Dokhi, Mohammad Al Naami, Syed Shahid Habib

**Affiliations:** 1 Muhammad Iqbal, Khalid A Al-Regaiey, Department of Physiology, College of Medicine & King Khalid University Hospital, King Saud University, P O Box 2925, Riyadh 11461, Saudi Arabia.; 2 Shafiq Ahmad, Department of Management, College of Business, Al-Yamamah University, P.O. Box 45180, Riyadh 11512, Saudi Arabia.; 3 Laila Al Dokhi, Department of Physiology, College of Medicine & King Khalid University Hospital, King Saud University, P O Box 2925, Riyadh 11461, Saudi Arabia.; 4 Mohammad Al Naami, Department of Surgery, College of Medicine & King Khalid University Hospital, King Saud University, P O Box 2925, Riyadh 11461, Saudi Arabia.; 5 Mohammad Al Naami, Department of Physiology, College of Medicine & King Khalid University Hospital, King Saud University, P O Box 2925, Riyadh 11461, Saudi Arabia.; 6 Syed Shahid Habib, Department of Physiology, College of Medicine & King Khalid University Hospital, King Saud University, P O Box 2925, Riyadh 11461, Saudi Arabia.

**Keywords:** Body composition, Bioelectrical Impedance Analysis, Body fat percentage, Physical fitness

## Abstract

***Objective: ***To determine an association between body composition analysis and physical fitness in the Saudi population and derive gender specific physical fitness equations.

***Methods: ***A total of 530 healthy Saudi adults aged 15-72 years (mean 37.16±14.12 years) were enrolled in this study. Body composition analysis was assessed by bioelectrical impedance analysis (BIA), with a commercially available body analyzer according to standard protocols.

***Results: ***Different body composition parameters, such as age, height, BSA (body surface area), obesity degree, body mass index (BMI), body fat mass (BFM) and percent body fat (% BF) contents were significantly different in males and females except weight which was non-significant (p=0.649). There was significant positive or negative correlation among different body composition parameters except weight with age in males and weight with age, height and BSA in females. In males, all the body composition characteristics contributed to the fitness score except BMI and BFM, while in females, the most significant effect was contributed by weight and BFM. Female body composition characteristics were strongly related to fitness score compared to males (R^2^ = 93.8% vs R^2^ = 78.5%).

***Conclusions:*** Different body composition parameters like BFM and %BF played an important role in determining physical fitness of healthy male individuals instead of BMI, weight and BSA, while in females weight was the best predictor of physical fitness.

## INTRODUCTION

Accurate body composition analysis predicts the physical fitness of human body and helps to understand the relationship among obesity, morbidity and mortality. Low physical fitness in adults is associated with different diseases such as type 2 diabetes mellitus, coronary heart disease, cardiovascular disease and even cancer.^1^^-^^4^ In children, low physical fitness is negatively associated with blood pressure and more prone to cardiovascular risk factors whereas in adolescents it can track to adulthood.^5^^,^^6^ Physical fitness is positively correlated with physical activity and has negative association with body fatness although this correlation is different in various ethnic background individuals.^7^^-^^9^ Improvement in physical fitness helps to prevent obesity and other related diseases. A systematic review of sedentary life style, particularly watching TV for more than 2 hours, of young children and youth suggests that it had negative effect on physical fitness and body composition and decreased academic achievement in school.^10^^-^^12^ When sedentary time period is reduced it had positive effect on body mass index (BMI).

The prevalence of obesity and related disorders are increasing in Saudi population^13^, however less information is available regarding the relationship of body composition with physical fitness. Data suggest that body composition parameters such as BMI and waist circumference (WC) analysis are very useful in predicting physical fitness and it has been shown to be negatively correlated with physical fitness.^14^ Different methods have been developed to measure body composition parameters. We used Bioelectrical Impedance Analysis (BIA), which is an easy, quick, cost-effective and painless test to determine body composition and fluid status and has been widely used in body composition analysis.^11^^,^^12^ In this method a non-detectable, safe low level current flows through the body. Total body water (72%) is the main component of the fat free body weight hence, BIA measures the resistance and impedance of current flows through extracellular and intracellular fluids, provided that body electrolyte status is normal.

Therefore, the primary aim of this study was to determine an association between body composition analysis and physical fitness for the Saudi population and secondary to investigate gender-specific difference in the physical fitness. Hence, a detailed assessment of different body composition parameters such as height, BSA (body surface area), weight, obesity degree, BMI, BFM kg (body fat mass kg), and % BF (per cent body fat) were taken into account when predicting the equation using BIA. We anticipate that this study would help in characterizing the prevalence of obesity and addressing future programs to control obesity and related ailments.

## METHODS

This cross-sectional study was conducted in the Department of Physiology, College of Medicine and King Khalid University Hospital, King Saud University, Riyadh, Saudi Arabia. A total of 530 healthy Saudi adults aged 15-72 years (mean 37.16±14.12years) were enrolled in this study which was approved by College of Medicine Ethics Review Board. Written consent was obtained from all the participants.

***Measurements of Anthropometric Indices and Body Composition Analysis: ***All participants underwent body composition analysis in the morning following an overnight fast and wearing light indoor clothes. Subjects were not allowed to drink during fasting and were asked to empty their bladders before measurements. Body composition was analysed by BIA, with a commercially available body analyzer (InBody3.0, Biospace, Seoul, Korea) as described previously^12^. The subjects were asked to first wipe the sole of the feet by a wet tissue and then stand over the electrodes of the machine and data were recorded in 3-5 minutes. Different body composition parameters, such as age, height, BSA, obesity degree, BMI, BFM, % BF contents and fitness scoring were based on the target values for ideal body fitness. BMI was calculated as the weight in kilograms divided by the square of the height in meters (kg/m^2^).

***Statistical Analysis: ***Statistical analysis was performed using IBM SPSS Statistics for Windows (version 20; SPSS Inc., Chicago, IL, USA) and Minitab version 15. Numeric data was summarized with mean, standard deviation (SD), median and range. Both gender groups (male and female subjects) were compared by using t-test for selected parameters. Pearson correlation analysis was conducted to determine interrelationship among different body composition parameters. Multiple regression models were further deployed to predict any significant relationship with fitness score of both male and female subjects. The analysis was carried out in different stages; first both groups were included with several characteristics of interest in the correlation study. This was then followed by including only the significant characteristics based on the observed p-values (the p-value of less than or equal 0.05 is considered to be significant). In the final stage we have conducted regression analysis for each group separately.

## RESULTS

Demographic analysis of all study subjects suggested significant differences among different body composition parameters such as age, height, BSA, obesity degree, BMI, fat mass, and %BF in both male and female subjects except weight which was non-significant (p=0.649, Table-I). Box plot comparisons between the characteristics of male (group1) and female (group 2) groups clearly showed that the fitness score for males was higher than females; however the SD for males was smaller than females. The median BMI was almost the same for both groups with higher SD for the females (data not shown).

Relationship between fitness score and other body composition characteristics for both groups, male and female subjects, were evaluated by correlation and multiple regression analyses. Correlation coefficients analysis illustrated that body composition parameters for male and female subjects were correlated positively or negatively with respect to different body composition characteristics significantly except weight with age (male subjects) and weight with age, height and BSA (female subjects) which were non-significant (data not shown).

The multiple regression model for male subjects (Table-II) with all predictors, independent variables, (R^2^ = 78.5%, F (10,352) = 128.614, P < 0.00) showed that all the body composition characteristics contributed to the multiple regression models and had significant effect to the fitness score except the BMI and BFM Kg. The regression model for the male subjects was as follows: The regression equation is: Fitness Score = - 156 - 0.0704 Age + 2.21 Ht - 56.5 BSA - 0.0072 Wt + 0.420 Obesity Degree + 0.098 BMI - 1.20 Fat Mass kg - 0.432 FAT - 0.652 Wt Cont + 0.622 Fat Cont

The multiple regression model for female subjects (Table-III) with all predictors, independent variables, (R^2^ = 93.8%, F (10,156) = 238.014, P < 0.00), showed that most significant effect was contributed by weight and BFM Kg whereas other body composition characteristics were not significantly contributing to the multiple regression model and did not have significant effect to the fitness score. The regression model for the female subjects was as follows: The regression equation: Fitness Score = 96.2 + 0.0646 Age - 0.065 Ht - 10.7 BSA + 0.758 Wt + 0.047 Obesity Degree - 0.47 BMI - 1.59 Fat Mass kg + 0.0506 FAT- 0.266 Wt Cont + 0.199 Fat Cont

Summary of both male and female regression models (Table-IV) suggested that female multiple regression model fits better than male (female R^2^ = 93.8% vs. male R^2^ = 78.5%).

## DISCUSSION

This is perhaps the first cross-sectional study to analyze body composition parameters and develop a gender specific physical fitness equation for healthy Saudi population using BIA. Pearson correlation and regression analysis indicated that almost all body composition parameters had significant interrelationships and there was a strong evidence that female regression model fits better than male (female R^2^ = 93.8% vs. male R^2 ^= 78.5%) indicating that female body characteristics were strongly related to fitness score than male subjects.

Regression analysis of data for female subjects showed that different body composition parameters did not contribute to the fitness score except fat mass and weight which had significant effect in predicting the fitness score. This can be explained due to physiological difference between men and women, as females have higher fat percentages and lower muscle mass. In a study of 14 years old school children, weight has significant effect on the physical fitness of the girls.^15^ Overweight girls had lower physical fitness and performed worst in physical fitness interventions than boys. A recent study from our laboratory has shown increased obesity prevalence in Saudi females compared to males (46.7% and 27.8% respectively, BMI≥30 kg/m^2^) and according to % BF, 64.7% and 57.9% respectively.^12^ Therefore, weight and fat mass are two important body composition parameters which have significant contribution in determining the physical fitness levels in Saudi female subjects.

**Table-I:** Comparison of gender differences in demographic data using T test

***Variable***	***Male***		***Female***		***P value***
	***Mean***	*** SD***	***Mean***	***SD***	
Age	38.763	15.563	33.689	9.488	0.000
Height	170.74	7.22	158.53	10.66	0.000
BSA	2.9204	0.2445	2.5245	0.2546	0.000
Weight	79.519	16.169	80.54	26.83	0.649
Obesity Degree	126.74	24.74	154.92	52.65	0.000
BMI	27.396	5.326	31.952	10.735	0.000
Fat Mass kg	22.167	10.311	35.2	20.57	0.000
% BF	26.843	8.004	40.369	11.192	0.000
Wt Cont	-3.962	14.801	-17.63	22.63	0.000
Fat Cont	-4.653	14.533	-18.42	22.01	0.000
Fitness Score	70.223	8.304	61.12	14.35	0.000

Wt Cont: weight control, Fat Cont: fat control

* Significant difference between male (n=363) and female (n=167) groups by 2 sample T Test (p < 0.05)

**Table-II:** Regression coefficients for predicting fitness using different body composition parameters (Male subjects).

***Independent Variables***	***Coefficients***	***T***	***P***	***95.0% Confidence Interval***	
				***Lower Bound***	***Upper Bound***
(Constant)	-155.748	-1.752	.081	-330.566	19.070
Age	-.070	-4.050	.000	-.105	-.036
Height	2.206	2.160	.031	.197	4.216
BSA	-56.461	-1.896	.059	-115.024	2.103
Weight	-.007	-.168	.866	-.091	.077
Obesity Degree	.420	7.936	.000	.316	.523
BMI	.098	.555	.580	-.250	.446
Fat Mass kg	-1.195	-10.775-	.000	-1.413	-.977
% BF	-.432	-4.407	.000	-.625	-.239
Wt Cont	-.652	-6.481	.000	-.849	-.454
Fat Cont	.622	6.133	.000	.423	.821
			
a. Dependent Variable: Fitness Score					
b. Selecting only cases for which Sex = male					

All the body composition characteristics contribute to the multiple regression model and have significant effect to the fitness score except the BMI, weight and BSA. The regression model for the male subjects is as follows:

The regression equation is:

Fitness Score = - 156 - 0.0704 Age + 2.21 Ht - 56.5 BSA - 0.0072 Wt + 0.420 Obesity Degree + 0.098 BMI - 1.20 Fat Mass kg - 0.432 %BF - 0.652 Wt Cont + 0.622 Fat Cont

**Table-III:** Regression coefficients for predicting fitness using different body composition characteristics (Female subjects).

***Independent Variables***	***Coefficients***	***T***	***P***	***95.0% Confidence Interval***	
				***Lower Bound***	***Upper Bound***
(Constant)	96.194	8.089	.000	72.703	119.684
Age	.065	1.857	.065	-.004	.133
Height	-.065	-.340	.734	-.445	.314
BSA	-10.735	-1.093	.276	-30.136	8.666
Weight	.758	4.655	.000	.436	1.079
Obesity Degree	.047	.255	.799	-.317	.411
BMI	-.470	-.407	.685	-2.757	1.816
Fat Mass kg	-1.594	-12.206	.000	-1.852	-1.336
% BF	.051	.608	.544	-.114	.215
Wt Cont	-.266	-1.949	.053	-.535	.004
Fat Cont	.199	1.491	.138	-.065	.463
a. Dependent Variable: Fitness Score					
b. Selecting only cases for which Sex = Female					

Most of the body composition characteristics are not significantly contributing to the multiple regression model and do not have significant effect to the fitness score except weight and fat mass kg. The regression model for the female subjects is as follows:

The regression equation is:

Fitness Score = 96.2 + 0.0646 Age - 0.065 Ht - 10.7 BSA + 0.758 Wt + 0.047 Obesity Degree - 0.47 BMI - 1.59 Fat Mass kg + 0.0506 %BF - 0.266 Wt Cont + 0.199 Fat Cont

**Table-IV:** Regression model summary for male and female subjects

***Model Summary *** a ***,*** b ***,*** c			
***Model***	***R Square***	***Adjusted R Square***	***Std. Error of the Estimate***
1. Male	.785	.779	3.9038
2. Female	.938	.935	3.6705

a . Predictors: (Constant), Fat Cont, Wt, BSA, Age, % BF, BMI, Fat Mass kg, Obesity Degree , Wt Cont, Height

b . Unless noted otherwise, statistics are based only on cases for which Sex :1= Male, 2= Female

c . Dependent Variable: Fitness Score

In males, most of the body composition parameters contributed in predicting fitness score, however, unlike females, parameters like BMI, BSA and weight, did not have any significant effect in predicting fitness level. BMI, subject’s height and weight ratio, is widely used as a measure of obesity and overweight as recommended by World Health Organization (WHO). It has been reported that BMI is not a good indicator of adiposity and underestimates excess body fat, therefore other body composition parameters should also be considered in assessing obesity and fitness level of individuals.^16^^-^^18^ There is a difference among BMI, % BF and body fat distribution parameters across populations.^19^ In certain ethnic backgrounds BMI is not an indicator of fatness/overweight, but represents muscle mass.^20^ A study of school-aged children had shown that BMI and waist circumference had negative correlation with physical fitness with more pronounced effects in older children.^14^ Data from our laboratory suggested that sensitivity of BMI to predict obesity was increased when its cutoff point was lowered from 27.5kg/m^2 ^(proposed for Asian population) to 26.6kg/m^2^.^12^ As discussed above, weight also did not have any significant effect in male subjects, although it played an important role in assessing the physical fitness levels of female individuals. Hence, body composition characteristics such as age, obesity degree, % BF and fat mass have significant role to predict physical fitness of Saudi male individuals instead of BMI, weight and BSA. 

The limitations to our study are the relatively small sample size and analysis of data from all age groups. Future study on large sample size and in different age groups would be useful to predict physical fitness equation and fitness levels in the population. We anticipate that this study would help to direct future health-related strategies and interventions to control obesity, type-2 diabetes and other health-related illnesses in the society.

## CONCLUSIONS

In our study different body composition parameters like fat mass, height, age, % BF, obesity degree, are important body composition parameters in determining physical fitness of healthy male individuals instead of BMI, weight and BSA. In females, only weight and BFM kg had significant effect in determining physical fitness instead of other body composition characteristics. Multiple regression models suggest that female body composition characteristics are strongly related to fitness score than male subjects.
